# Assessment of Countries’ Preparedness and Lockdown Effectiveness in Fighting COVID-19

**DOI:** 10.1017/dmp.2020.217

**Published:** 2020-06-24

**Authors:** Faten Amer, Sahar Hammoud, Bashar Farran, Imre Boncz, Dóra Endrei

**Affiliations:** Doctoral School of Health Sciences, Faculty of Health Sciences, University of Pécs, Pécs, Hungary; Faculty of Modern Philology and Social Sciences, University of Pannonia, Veszprém, Hungary

**Keywords:** COVID-19, forecast, ICU capacity, lockdown, pandemic

## Abstract

**Objectives:**

The aim of this study was to assess the risks in confronting the coronavirus disease 2019 (COVID-19) pandemic and the ongoing lockdown effectiveness in each of Italy, Germany, Spain, France, and the United States using China’s lockdown model simulation, and cases forecast until the plateau phase.

**Methods:**

Quantitative and qualitative historical data analysis. Total Risk Assessment (TRA) evaluation tool was used to assess the pre-pandemic stage risks, pandemic threshold fast responsiveness, and the ongoing performance until plateau. The Infected Patient Ratio (IPR) tool was developed to measure the number of patients resulting from 1 infector during the incubation period. Both IPR and TRA were used together to forecast inflection points, plateau phases, intensive care units’ and ventilators’ breakpoints, and the Total Fatality Ratio.

**Results:**

In Italy, Spain, France, Germany, and the United States, an inflection point is predicted within the first 15 d of April, to arrive at a plateau after another 30 to 80 d. Variations in IPR drop are expected due to variations in lockdown timing by each country, the extent of adherence to it, and the number of performed tests in each.

**Conclusions:**

Both qualitative (TRA) and quantitative (IPR) tools can be used together for assessing and minimizing the pandemic risks and for more precise forecasting.

It has been more than 2 mo since China has imposed a lockdown on January 23, 2020, as an attempt to limit the spread of the novel coronavirus disease 2019 (COVID-19) any further. Other countries have followed this step after the World Health Organization (WHO) announced COVID-19 as a pandemic as of March 12, 2020. Most countries initiated a travel ban for travelers. However, scientists consider that this intervention may have only postponed the spreading of the virus for another 3 to 5 d.^1^ We think that social distancing precautions are vital in fighting the COVID-19 pandemic, but they must be evaluated through periodical assessments to maintain their effectiveness.

Remuzzi and Remuzzi^2^ predicted the number of COVID-19 cases and the number of intensive care unit (ICU) patients in Italy until March 16, 2020, which was only 1 wk after lockdown initiation. Pueyo^1^ made a forecast for the new number of cases depending on the previous trend of increase up to March 14, 2020, which also preceded the date at which most of the European countries took the decision of lockdown. Up to our knowledge, there is no study that combines historical data with the country’s specific characteristics and responses types in forecasting; which means analysis of both quantitative and qualitative data together, instead of depending solely on 1 of them.

This study is one of the earliest studies that assess countries preparedness, through developing a risk evaluation tool to evaluate strengths and weaknesses for countries in confronting COVID-19. WHO^3,4^ referred partially to this concept even if not in a comprehensive methodology as in this study. Other researchers as Gilbert et al.^5^ used the State Party Self-Assessment Annual Reporting tool (SPAR),^6^ which was developed also by the WHO in 2018 and contains 24 indicator scores for the 13 International Health Regulations (IHR) capacities needed to detect, assess, notify, report, and respond to public health risk and acute events of domestic and international concern. This tool was not specifically developed for the preparedness assessment against COVID-19 but for general assessment of countries’ preparedness using their annual reports. Mei and Hu^7^ and Gemelli^8^ analyzed reports of COVID-19 published by the WHO for the Western Pacific Islands and Argentina, respectively. These studies analyzed only the data of total cases, deaths, and the time of the first infection report. While, James^9^ discussed testing and risk factors in general. On the other hand, Ali^10^ analyzed COVID-19 second wave preparedness depending on the Spanish flu pandemic as well as infodemics. For these reasons, we believe that there is a crucial need to develop a preparedness assessment tool that is customized specifically to analyze the preparedness for COVID-19.

Moreover, to our knowledge, it is mostly the first research that aims at estimating the average Infected Patient Ratio (IPR) who is infected by 1 primary infector in the incubation period, while considering changes in this ratio as evidence for evaluating social distancing or lockdown effectiveness. Most importantly, it aims to forecast new cases and deaths in Italy, Germany, Spain, France, and the United States, not only by simulating it to China’s quantitative model but also by considering other qualitative vital indicators, such as lockdown timing, adherence to it, the number of performed tests, and initiatives done to lower the deficits in ICU capacity. The combination of both quantitative and qualitative data in rendering the forecast can lead to more precise estimations.

## METHODS

### Total Risk Assessment Tool

Initially, the selection of countries in this study was based on the 6 countries with the highest observed cases until April 4, 2020, and with the highest transparency and accuracy in case disclosure. In this research, 2 evaluation tools were developed. The first evaluation tool focused on the Total Risk Assessment (TRA) score for each country through several indicators at each stage.

The first indicator is the total population, that is, countries with high total population are expected to have more cases and deaths. This indicator cannot be avoided and is vital to be considered among the risk indicators. The second indicator is the population’s density, which makes social distancing more difficult. The third indicator is the age distribution, i.e., the risk becomes higher for those countries that have a higher percentage of elderly people (older than 65 y old). Per the Centers for Disease Control and Prevention (CDC),^11^ 31% to 64% of COVID-19 patients who are 65 y old and older will require hospitalization. Regarding the fourth and fifth indicators, the risk elevates for those countries that have lower numbers of ICU beds per capita, or lower ventilators per capita. Although both indicators are interconnected, it is essential to evaluate them independently.^12^ The deficit compensation for these 2 indicators should be evaluated also at each stage.

The sixth indicator is the lockdown timing evaluation. The interpretation of lockdown timing in this study is assessed by calculating the ratio of cases per capita at the time of lockdown. The importance of this indicator emerges from the idea that each day of delay in announcing social distancing precautions makes the total cases peak earlier and higher.^1^ It is also important to check the population adherence to lockdown measurements as well. The seventh indicator is the number of performed tests per million capita. Countries with higher rates of confirmed cases tend also to be the countries where a larger share of their population has been tested, but it does not actually mean that these countries have more cases than those who are not performing testing or have underestimated cases. Tracking cases will pay off later by lowering the exponential growth in the long run, even if it increased for some time.

These indicators have been used to evaluate the TRA during 3 stages in each country using a 5-point scale for each indicator, where 1 is the lowest risk and 5 is the highest. The first stage (Pre-pandemic Stage) was evaluated using the first 5 risk indicators for preparedness assessment, with a TRA scale of 1-25. In the second stage (Pandemic Threshold Stage), the lockdown timing evaluation has been added to the previous 5 risk indicators to evaluate the response with a 1-35 TRA scale. In the third stage, (Post-threshold Stage), the number of COVID-19 performed tests indicator has been introduced to assess the number of performed tests from the last stage up to the latest available data. An update for solving the deficits in the fourth and fifth indicators was required in the last 2 stages. The sum of the 8 indicators has been used to assess the last stage with a TRA scale of 1-40. Stage 3 evaluation has been made until April 3, 2020. Finally, all TRA scales’ results were converted out of 100 to ease the comparison process of TRA scores (see the online Supplemental Appendix 1).

### IPR Tool

Regarding the second evaluation tool, the IPR was measured by calculating the number of cases resulting from 1 primary infector during the incubation period. In China, for example, the IPR dropped from 38 infected patients to 4 after only 12 d of lockdown. This ratio dropped again to 1 patient after another 10 d. The trend’s change in total cases is difficult to notice before arriving at an inflection point, while it is possible to notice the IPR change from the first 10 d of lockdown. The intervals for calculating the IPR were selected to be in coherence with the incubation period; the period between the exposure to an infection and the appearance of the first symptoms. According to the CDC,^11^ the incubation period is 2 to 14 d, although per the WHO,^13^ it is between 2 and 10 d. Consequently, 10 d were chosen to be considered for estimation. Therefore, the following tool for IPR measurement was developed as the formula:
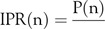


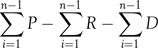
where IPR(n) is the number of patients in the interval (n) who were infected by 1 primary source in the past 10 d. Meanwhile, this primary source can be any active patient from an earlier interval who was still an active infector during the interval (n − 1). Therefore, patients who either recovered or died before or during the interval (n − 1) were excluded. P(n) is the total observed new patients in the interval*study*(n). While 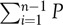 is the total observed patients in all the previous intervals excluding both 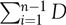, which is the total of patients who have died until the end of the interval *study*(n − 1), and 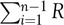, which is the total recovered patients until the same interval.

The IPR tool has been used to evaluate the lockdown effectiveness in each country as well as building the forecast, taking into consideration the differences in population’s adherence to lockdown among countries, which has been categorized to: loose lockdown adherence, intermediate lockdown adherence, strict lockdown adherence. Moreover, the rapidness of testing increase differences has been categorized into: rapid testing increase; intermediate testing increase, slow testing increases categories (see Table 1).

**TABLE 1:** Observed and Simulated IPR Values, Stratified by Incubation Intervals (Int. = 10 Days)

Int.	Italy	Int.	Spain	China	Germany	France	USA	Stage	IPR 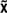 /stage
Int. 1	**0**	Int. 1	**0**	**0**	**0**	**0**	**0**	Pre-intervention	16
Int. 2	**15.38**	Int. 2	**93**	**3.4**	**76**	**46**	**3**		
		Int. 3	**27**	**38**	**6**	**8**	**16**		
Int. 3	6.3 ^>*^	Int. 4	**5.78 ^#’^**	**4 ^<’^**	8 ^#*^	**6 ^#’^**	**18 ^>’^**	**LD**	**6**
Int. 4	2.41 ^>*^	Int. 5	**1.89** ^**#**^ *”*	**1.13** ^*<*^ **’**	**2** ^#*^	**3.6** ^**#’**^	**3.6** ^**#’**^	Post-intervention	
Int. 5	**0.87**	Int. 6	*0.62*	**0.08**	*0.6* ^#*^	*0.7* ^**#**^ *”*	*1* ^**#**^ *”*		Depends on:
Int. 6	*0.4*	Int. 7	*0.31*	**0.04**	*0.17*	*0.27*	*0.5*		1. The interval sequence
Int. 7	*0.25*	Int. 8	*0.18*	**0.01**	*0.08*	*0.18*	*0.27*		2. LD timing
Int. 8	*0.16*	Int. 9	*0.12*	**0.03**	*0.04*	*0.12*	*0.17*		3. LD adherence
Int. 10	0.08	Int. 10	*0.09*	**0.02**	*0.02*	*0.8*	*0.11*		4. Testing numbers increase
Int. 11	0.05	*Int. 11*	*0.06*	*0*	*0.01*	*0.05*	*0.08*		
Int. 12	0.02	*Int. 12*	*0.05*	*0*	*0*	*0.02*	*0.06*		
Int. 13	0.01	*Int. 13*	*0.03*	*0*	*0*	*0.01*	*0.05*		
Int. 14	0	*Int. 14*	*0.2*	*0*	*0*	*0*	*0.04*		
Int. 15	0	*Int. 15*	*0.1*	*0*	*0*	*0*	*0.03*		
		*Int. 16*	*0*	*0*	*0*	*0*	*0.01*		

Int., interval; LD, lockdown; ^>^, loose lockdown adherence; ^#^, intermediate lockdown adherence; ^<^, strict lockdown adherence; *, rapid testing increase; ^”^, intermediate testing increase; ’, slow testing increase. Bold are Observed IPR, *Italic* are simulated IPR.

The median of IPR at the observed points has been calculated then simulated to estimate other countries’ IPR while taking the TRA score of each country into consideration. A forecast for the inflection point, plateau, ICU capacity breakpoint, ventilator deficit, and Total Fatality Ratio (TFR) has been projected based on these 2 tools (see Supplemental Appendix 5).

For countries that will stay behind the ICU breakpoint capacity, the TFR was calculated by multiplying the total estimated cases for each country by the historical TFR at the last interval to avoid the bias of underestimated TFR at the early stage of the pandemic (a.k.a. Case Fatality Rate). It was more challenging to calculate TFR for those countries which are expected to exceed their ICU capacity; the estimated deficits should be turned into estimated deaths. China has reported that 5% of COVID-19 patients need ICU admission, while in Italy researchers reported that the percentage was 12%.^14^ China’s reported percentage was used as a reference point for the other countries excluding Italy. Moreover, COVID-19 patients need an average of 10 d at the ICU.^15^ So, an average of 10 d was used in this research for ICU beds and ventilators estimation. Regarding estimations of the number of free beds, the Organization for Economic Co-operation and Development (OECD)^16^ mentioned that the general hospital occupancy rates were ranging from 65% to 80%, but no occupancy rates were specified for ICU beds. Therefore, to avoid an overestimation of the free beds needed, the ICU occupancy rates were considered as the following: 90% in China due to the low number of ICU beds per capita, 70% for each of Germany and the United States, and 80% in other European countries. The estimated TFR is calculated using 2 methods. TFR_1_ is calculated by adding the adjusted TFR in intervals at ICU breakpoints to the last recorded TFR before breakpoints. TFR_2_ is calculated by considering the historical death rate in the closed cases and performed tests number evaluation indicating the volume of underestimated cases, which means using TRA and IPR tools to estimate TFR_2_.

## RESULTS

Regarding the first and second risk indicators, all of Italy, Germany, Spain, and France have an intermediate total population and density in comparison to the United States and China.^17^ Concerning the third indicator (elderly percentage), 11%, 23%, 21%, 20%, 19%, and 16% of populations were older than 65 y old in China, Italy, Germany, France, Spain, and United States, respectively (see Supplemental Table 1.1).^18,19^ Per the fourth indicator, the ICU beds ratio per 100K capita was evaluated as a measuring unit at the Pre-pandemic Stage to assess the preparedness for each country. China’s ratio was only 3.6 beds.^20^ Whereas, in all of Spain, France, and Italy it was between 10 and 13 beds. On the other side, both Germany and the United States had ratios higher than 29 beds^21,22^ (see Supplemental Table 1.1). Tackling the fifth indicator, on March 15, 2020, the United States has increased the number of ventilators to become 160 K,^23^ while Germany initially had 25 K ventilators in the Pre-pandemic Stage.^24^ However, Italy and France had only 3 K and 4.5 K ventilators, respectively.^25,26^

On the other hand, it was not possible to find any disclosed information that refers to the number of ventilators either in Spain or in China. In the sixth risk indicator, an evaluation of lockdown timing has been made using the ratio of confirmed cases per 100K capita at the time of the lockdown announcement. This ratio was 0.3 for China at that time.^19,27^ On the contrary, it was 8.7, 10.2, 12.2, and 13.7 for Germany, France, Italy, and Spain, respectively.^19,27^ For the United States, the lockdown has not been initiated until March 22, 2020, when this ratio was estimated as 10.2 at that time. Regarding the seventh risk indicator, up to March 15, 2020, each of Italy, China, and Germany performed more than 2 K tests/1 M capita,^19,28^ with the highest ratio for Germany. On the contrary, the ratio in each of France and the United States was less than 600, with the lowest ratio for the United States,^19,28^ which indicates underestimated total cases at that time. While at the end of March, Spain increased this ratio to more than 7 K,^19,29,30^ whereas Germany and Italy raised it to more than 10 K each. On the other hand, France and the United States raised it to more than 3.6 K tests each. Moreover, the United States has enormously increased this ratio per selected state as in New York’s ratio to arrive at 14 K.^31^

**FIGURE 1:**
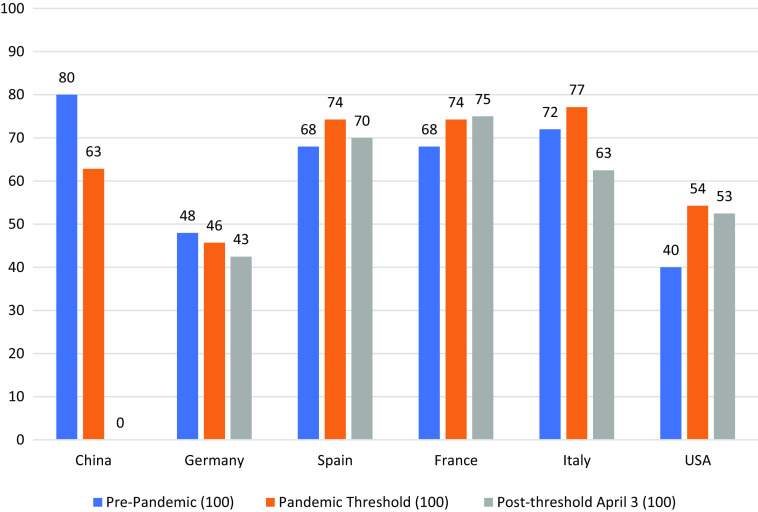
**Fluctuations of TRA Scores Per Stage.** TRA scores per 3 stages: first stage, pre-pandemic stage; second stage, pandemic threshold stage; third stage, post-threshold stage on April 3, 2020; score (1-100), where 100 is the highest TRA, while 1 is the lowest TRA.

As a final result, TRA in the third stage for all of Italy, Germany, Spain, France, and the United States was 63%, 43%, 70%, 75%, and 53%, respectively. This TRA was taken into consideration in the next IPR forecast (see Figure 1). Based on IPR, a drop has been noticed immediately after lockdown for all countries (see Supplemental Table 1.2). It has been noticed that the IPR median for all countries*study* in the intervals before the lockdown was 16. In the first 10 d of lockdown, it dropped to 7 patients, while in the next 10 d, it dropped to 2 patients.

## DISCUSSION

### TRA Tool

China’s and the United States’s total population and density put both countries at higher risk compared with the European countries, especially in some states such as New York due to the higher population density. Having a higher total population means that the upper limit of the possible total cases is going to be bigger, while higher population density makes the social distancing difficult to achieve, as in the United States or China. Regarding the age indicator, Italy and Germany are considered at the highest point in the risk ladder for this indicator analysis, followed by the United States, Spain, and France, while China is at the lowest point.

For the number of ICU beds per 100 K capita-risk indicator, the evaluation was supposed to impose an extremely high risk on China unless a fast response has been performed. Meanwhile, Italy, Spain, and France are in a moderate position per this risk indicator. Whereas, Germany and the United States are considered in the lowest risk position due to the high number of ICU beds, while it is still essential to be considered for each state or city alone, especially if the cases are not spread in a homogeneous pattern, such as the case of the United States where 40% of cases are in New York until the beginning of April. This means that 40% of the needed ICU beds are going to be needed there, which may lead to a breakpoint and hence higher deaths than expected even if the total number of beds in the United States shows no capacity break.

On the other hand, the low number of ventilators in Italy and France, and the unavailability of any disclosed information regarding this indicator in China and Spain are supposed to put all of them under great risk in this crisis. However, attempts in the United States to increase the ventilators at later stages will lower the TRA, and this is why it is important to perform a continuous update for the fourth and fifth indicators at each interval.

Regarding the lockdown indicator, some researchers consider that China was late in announcing the lockdown,^1^ but according to the number of cases per capita at the time of lockdown, it can be considered early timing compared with the other countries, which in fact led to a faster containment of the virus, fewer cases, and deaths in China. Moreover, it is vital to frequently update the number of performed tests to be evaluated in TRA. Germany and Italy can be considered at lower risk position per this indicator due to the high number of performed tests by both countries, while in the United States and France, it is the opposite that put them on the highest risk per this indicator. However, the latest attempts of the United States to increase the number of performed tests should be taken into consideration for future forecasts. Whereas France is expected to be the slowest in arriving at a plateau if the number of performed tests is not increased any further, because according to TRA, it is assumed that the current number of total cases is underestimated, and this can be the explanation why France has a higher number of deaths per total cases compared with other countries.

By combining the evaluation of all the previously mentioned indicators, the TRA of China was found to be the highest in the first stage among all others, followed by each of Italy, France, and Spain. While for the United States and Germany, TRA was the lowest. Despite that, China was able to lower the TRA significantly in the second stage due to the early lockdown timing and the strict adherence to it. This led China to arrive at a plateau rapidly without even the need of performing a high number of tests, although this may lead to underestimation of infected total cases as well as total deaths. Whereas the TRAs for all the other countries have decreased in the next stage due to the late lockdown announcement, which coincided with a deficit in meeting the massive demands on tests and ICU beds, Germany performed better in these indicators.

The added factors for assessing the third stage, namely the number of tests and factors 3 and 4 updates led to different results for each country. Therefore, despite the low TRA already in Germany in second stage, in the third stage, Germany’s TRA score decreased even more but slightly. As for Italy, the decrease in TRA was sharp due to increases in the mentioned factors. Moreover, Spain and the United States had a slight decrease as well because of partially solving the factors issues. Meanwhile, the initiative of France to increase the number of performed tests per million capita was the slowest among all countries until the fifth interval on April 4, 2020 (see Figure 1). This prevents France from decreasing the TRA score at the third stage. However, in the United States scenario, customization for each state alone should be considered in future research to unravel the image that reflects the real status quo. Additionally, the low performed tests at the time of lockdown indicate underestimated total cases.

### IPR Tool

The forecast in Table 2 was simulated, depending on both TRA and IPR tools. As noticed, the drop in IPR means that the lockdown has started to be effective from the first day in all countries, but varied from 1 country to another. The IPR was simulated after lockdown and was based on 3 factors: the extent of adherence to lockdown, the increase in performed tests, and the interval sequence (see Table 2). The biggest drop in the IPR tool is expected to be in China due to the early announcement of the lockdown and the strict adherence to it. On the contrary, the smallest drop in the interval following lockdown was in the United States and France, which resulted from the late lockdown timing and the loose adherence to its measures in both countries. Meanwhile, the drop in future IPR is expected to be also the lowest in France and the United States due to the slow increase in testing number per capita, even if the total number of tests was high. As a result, it is expected to have a higher number of deaths in countries with a low number of tests due to the underestimation of true cases there.

**TABLE 2:** Evaluation and Approximate Forecast Until May Using TRA and IPR Tools

Tool	Perspective/Country	China	Italy	Germany	Spain	France	USA
	TRA1 (pre-pandemic)	76	68	36	56	60	28
	TRA2 (pandemic threshold)	70	87	43	77	80	53
TRA	TRA3 (post-threshold)	Finished	94	49	89	100	69
	(TRA1 to TRA3)	(-)	(+/-)	(+/-)	(+/-)	(+/+)	(+/+)
	Est. Total cases	86K	267K	171K	333K	253K	2M
	Est.total Deaths 1	3.5K	27K	2.2K	20K	16K	51K
	Est.total Deaths 2	7K	81K	7K	90K	87K	358K
IPR	Est.TFR 1	4.1%	10.5%	1.3%	6.0 %	6.2%	2.5%
	Est.TFR 2	8.1%	31.4%	3.9%	26.9%	34.3%	17.4%
	Est.ICU total deficits*	0**	17K*	0**	7.6K*	2K*	0**
	Est.breakpoint period	Non	Mar. 6- May.14	Non	Mar.16- Jun.3	Mar.26- Apr.24	Mar.26 –May.24
	Est.inflection P. (IPR<1)	15-Feb	02-Apr	11-Apr	11-Apr	11-Apr	11-Apr
IPR	Est.days since LD	20 days	26 days	27 days	29 days	27 days	21 days
IPR	Est.plateau P. (IPR=0.08-0.04)	05-Mar	15-May	10-May	13-Jun	04-Jun	03-Jul
	Est.days since LD	39 days	79 days	51 days	92 days	81 days	104 days

Est., estimated; F., finished; LD, lockdown; P, point; TRA, Total Risk Assessment; TRA1, TRA in Pre-pandemic Stage; TRA2, TRA in Pandemic-Threshold Stage; TRA3, TRA in Post-threshold Stage.

* Cases of ICU beds capacity increased (a portion of this number will not die).

** Only in certain states or cities if cases distribution is not uniformed in the country (eg, New York, calculations need to be reconsidered for each state, city independently).

We believe that this study has several strengths. First, it offers an easy approach for countries to evaluate their TRA at all pandemic stages. Second, this article builds a forecast for the new cases, deaths, and TFR estimation after considering ICU capacity breakpoint instead of depending on the historical TFR alone, as well as considering the death rate in closed cases and the underestimated cases. Third, combining the use of IPR and TRA makes the forecast more efficient and comprehensive.

On the other hand, the 2 evaluation tools have some limitations. First, TRA does not include the assessment of other indicators, such as medical supplies availability (medications, masks, and antiseptics), as well as the medical human resources capacity. Second, TRA was assessed based on the available number of performed tests per million capita until April 4, 2020, during 3 intervals. Therefore, it is recommended to update all of TRA, IPR, and the forecasting built based on them periodically. Third, in our forecast using the IPR tool, the percentage of patients who need ICU admission, the ICU occupancy rate, and the incubation period of COVID-19 were estimated per the references.^11,13,14,16^ Fourth, the evaluation of public lockdown adherence has been made according to the available news from each country. Finally, the use of the estimated 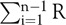 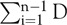, as part of the IPR forecast formula denominator can result in a small margin of error. Moreover, to have more precise results it is essential to re-apply this methodology for each city, state, or province by governments if the following factors were not spread homogeneously at that country: the total population, population density, the number of tests, the number of ICU beds, the extent of lockdown adherence, the total cases, the deaths, the recoveries or any of them, which was not possible to be performed in this study due to the lack of data.

Finally, the seasonal effect on cases number as well as the re-opening effect was not taken into consideration in this forecast; these factors are recommended to be analyzed by other researches. These theoretical models would benefit from being tested and verified using statistical analysis based on observed data once available. Based on the statistical analysis data afterward, different weights can be assigned to each factor reflecting its importance.

## CONCLUSIONS

A median of 16 patients was infected by a primary infector during the incubation period in all 6 countries. The TRA tool can be used as both planning and/or evaluation tool for countries. By using the IPR tool, a spontaneous decrease after 10 d of lockdown can be noticed if the lockdown is effective. The earlier announcement of lockdown and the stricter the adherence can lead to fewer infected total cases, fewer deaths, and faster to a plateau, a more precise estimation of cases, and fewer deaths. Consequently, leading to a faster containment of the virus and mitigate consequences.

The estimated cases in the United States using the IPR and TRA tools are higher than those predicted by the White House,^32^ Institute for Health Metrics and Evaluation (IHME),^33^ and Dr. Fauci.^34^ Even if COVID-19 total tests were increased dramatically lately there, it is still evaluated as an intermediate number per capita when compared with other countries, such as Germany, which has increased the number of testing at an early stage. It is meaningless to evaluate the lockdown timing according to the number of days since the appearance of the first case; rather it should be evaluated by the number of cases per capita at the time of lockdown. However, it is essential to consider the number of testing as well, because having a low testing number at the time of lockdown may hide underestimated total cases that were not tested; therefore, a later lockdown decision at that point could be considered late, such as in the case of the United States.

In closed cases, death rate was the highest in the United States, Italy, France, Spain, and Germany, respectively, until April 4, 2020. Total cases and death rates in the United States were supposed to be much less if the lockdown was announced earlier, and if the number of tests was increased faster and earlier than March 2020, and the same applies for Spain and France. However, although China took the lockdown decision early and strictly adhered to it, the total cases and deaths there could be underestimated due to the low number of testing. Whereas, although Germany is considered late in comparison to China’s case in taking the lockdown decision, it is still earlier than other countries’ decisions, which coincided with a faster increase in testing and made it easier to control the pandemic and to arrive at a plateau faster. Italy could have had fewer cases if the adherence to lockdown was earlier and stricter; moreover, the elderly percentage played a vital role in increasing the death rate there. In general, countries where the decision of lockdown was taken earlier by their decision-makers, with better adherence to it, were able to lower the total cases and the total deaths. Meanwhile, countries that increased the number of testing substantially at an early stage were able to arrive at a plateau faster, hence minimizing the case and death numbers eventually, as well as having a better estimation of these numbers.

There is no apparent reason for the TRA, IPR, and TFR tools proposed in this research not to be generalized or replicable to other countries’ scenarios in controlling the COVID-19 pandemic as long as reliable data are available. Moreover, it is advised to use IPR and TRA tools together for better management of this pandemic and more accurate forecasting. However, it is advised that another assessments be made for these countries after arriving at a plateau point to draw lessons from the past in confronting future pandemics. Finally, these 2 tools can be used in other pandemics’ cases with some modifications.

## Supplementary material

For supplementary material accompanying this paper visit https://doi.org/10.1017/dmp.2020.217. click here to view supplementary material
